# Acute Pancreatitis Likely Due to Semaglutide

**DOI:** 10.7759/cureus.69844

**Published:** 2024-09-21

**Authors:** Katie Hughes, Yovan Ram Kurrun Sumaruth, Elmahi Mohammed, Vibhootee Sant Bakshsingh

**Affiliations:** 1 Medicine, North Manchester General Hospital, Manchester, GBR; 2 Internal Medicine, North Manchester General Hospital, Manchester, GBR; 3 Internal Medicine, Royal Gwent Hospital, Newport, GBR

**Keywords:** semaglutide glp-1 analogue, lipase, obesity, glp-1 receptor agonists, semaglutide, acute pancreatitis

## Abstract

Semaglutide is a glucagon-like peptide-1 receptor agonist (GLP-1RA) that has gained recent popularity in its effective management of type 2 diabetes mellitus (T2DM) and obesity. Minimal evidence has reported the link between Semaglutide use and acute pancreatitis. In this case report, we discuss the case of a 36-year-old female presenting to the Emergency Department with sudden-onset epigastric pain, subsequently diagnosed with acute pancreatitis. Moreover, she had recently started subcutaneous semaglutide injections for weight loss, which she had procured from one of her acquaintances without seeking medical advice. Semaglutide was thus stopped and her lipase levels normalized with significant improvement of her symptoms, making semaglutide the likely causative factor for her acute pancreatitis. Given the increased use of GLP-1RA, we aim to increase awareness among patients taking this medication whether prescribed or not and increase clinician awareness when prescribing this medication.

## Introduction

Semaglutide operates as a glucagon-like peptide-1 receptor agonist (GLP-1RA), sharing a remarkable 94% structural homology with one of the incretin hormones, human glucagon-like peptide-1 (GLP-1) [1]. GLP-1RAs were initially developed for the treatment of type 2 diabetes mellitus (T2DM) [1]. The mechanism through which semaglutide delivers its benefits in lowering blood glucose levels involves the activation of GLP-1 receptors primarily located in the gastrointestinal (GI) tract, pancreas, and brain [1]. Upon activation of the GLP-1 receptor, semaglutide enhances glucose-dependent insulin secretion, providing a physiological response to elevated blood glucose levels after a meal [2,3]. Simultaneously, it slows gastric emptying, increases pancreatic β-cell proliferation, and reduces glucagon release, contributing to an overall reduction in appetite thus also promoting weight loss [1-3]. In addition, trials have shown semaglutide to be efficacious in reducing the rate of non-fatal myocardial infarction (MI), non-fatal cerebrovascular events, and cardiovascular death in T2DM patients [4]. This led to an update in the guidelines from the American Diabetes Association and the European Association for the Study of diabetes to recommend the use of semaglutide in any diabetic patient with or at increased risk of cardiovascular disease [5].

Given this vast range of positive health benefits, in June 2021, the Food and Drug Administration (FDA) expanded the clinical indication of semaglutide to include weight management in adults who are obese or overweight [6]. Thus, semaglutide is now the active ingredient in the well-known obesity-licensed medication Wegovy, licensed in the US and UK in 2021 and 2024, respectively [7]. As a result, the use of semaglutide in obesity management is relatively novel, and like any novel drug, unexpected and potentially harmful side effects can be uncovered with more widespread use. Commonly listed adverse effects of semaglutide include GI discomfort, diarrhea, cholelithiasis, and hypoglycemia [8]. Notably, the risk of developing acute pancreatitis has been reported with semaglutide use; however, recent studies suggest that there is no increased risk of acute pancreatitis with subcutaneous semaglutide use compared with non-users [9,10]. In this report, we discuss a case of a patient who developed acute pancreatitis likely secondary to semaglutide use.

## Case presentation

A 36-year-old woman with a background history of anxiety, depression, chronic back pain, and previous cholecystectomy (approximately 10 years ago) presented to the Accident and Emergency department with a one-day history of sudden onset epigastric pain. The pain was described as a severe sharp pain radiating into her back, initially brought on at rest. The pain was associated with nausea and she reported multiple episodes of watery diarrhea in the week prior to presentation. She denied any other symptoms. The patient had been commenced on once-weekly semaglutide injections for weight loss five weeks prior and her last dose of semaglutide had been in the week prior to presentation. She procured the medication from one of her acquaintances without a prescription and was not aware of the dose she was taking. She was not assessed by a medical professional prior to taking this medication. Her other long-term medications included mirtazapine (started in February 2024) for anxiety and depression, amitriptyline (started in May 2020), and pregabalin (started in May 2021) for back pain. All of these medications had remained unchanged. She denied smoking or recreational drug use and drank on average 10-15 units of alcohol per week. She denied an increase in alcohol consumption in the weeks prior to the presentation. Vitals on admission were temperature, 36.7 °C; heart rate, 101; respiratory rate, 18; blood pressure, 132/79 mm Hg; and saturation, 98% on room air. On examination, her abdomen was soft with epigastric tenderness in the absence of guarding and her bowel sounds were present. Admission blood revealed a lipase of 1367 U/L (normal reference: 13-60 U/L), alanine transaminase of 64 IU/L (normal reference: 1- 35 IU/L), bilirubin of 6 umol/L (normal reference: 0-21 umol/L), alkaline phosphatase of 64 U/L (normal reference: 30-130 U/L), adjusted calcium of 2.26 mmol/L (normal reference: 2.20-2.60 mmol/L), and triglycerides of 1.5 mmol/L (normal reference: 0.0-1.6 mmol/L) (Table 1).

**Table 1:** Admission blood results ALT, alanine transaminase; ALP, alkaline phosphatase

Investigation	Value	Reference range
Lipase	1367 U/L	13-60 U/L
ALT	64 IU/L	1-35 IU/L
Bilirubin	6 umol/L	0-21 umol/L
ALP	64 U/L	30-130 U/L
Adjusted calcium	2.26 mmol/L	2.20-2.6 mmol/L
Triglycerides	1.5 mmol/L	0.0-1.6 mmol/L

**Figure 1:**
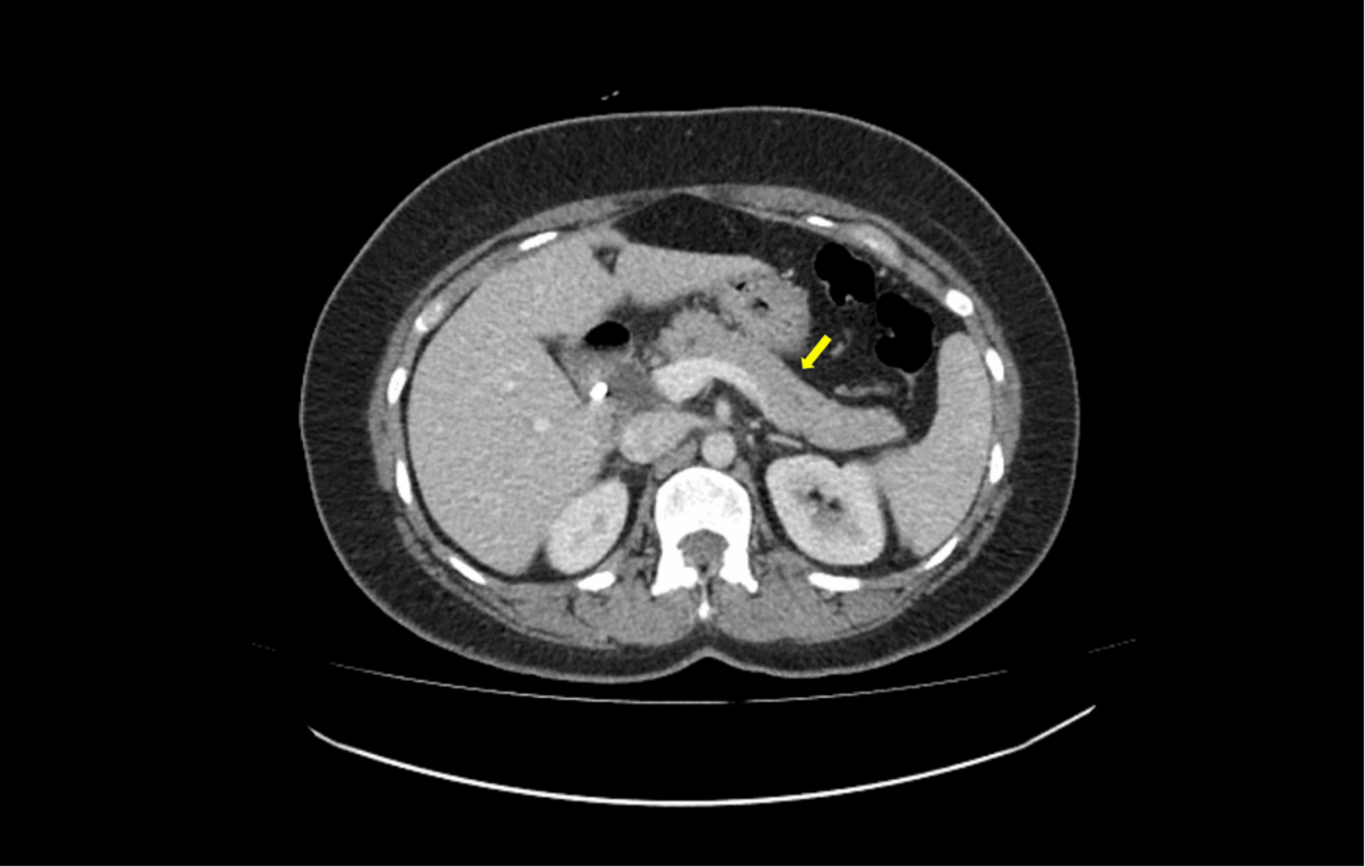
CT abdomen and pelvis with contrast There is normal enhancement of the pancreas with no CT evidence of acute pancreatitis or peripancreatic collections, and no other acute intra-abdominal pathology identified. The yellow arrow indicates the pancreas.

Feces cultures were sent due to ongoing diarrhea, all of which were negative. Hepatitis B and C and HIV tests were also negative. The IgG4 subclass was not elevated. CT abdomen and pelvis with contrast showed normal enhancement of the pancreas with no CT evidence of acute pancreatitis or peripancreatic collections, and no other acute intra-abdominal pathology identified (Figure 1).

The patient was reviewed by gastroenterology who advised an ultrasound abdomen, as a CT scan is unlikely to show early changes in the pancreas if symptom onset is less than 72 hours. On the fourth day of admission, the patient had an ultrasound abdomen with the following report: the pancreatic head was normal, the body and tail were obscured, and unremarkable ultrasound appearances. Despite normal imaging, the patient was treated for acute pancreatitis attributed to semaglutide. It is worth noting that mirtazapine has been associated with pancreatitis secondary to raised triglyceride levels. However, given that this patient had normal triglyceride levels, it is unlikely that mirtazapine is the cause of pancreatitis in this case [11]. In addition, given her minimal alcohol intake, negative relevant investigations, and no other relevant medications, the most likely cause of her pancreatitis was semaglutide. Thus, her semaglutide was discontinued and she was discharged home with a resolution of her symptoms. 

## Discussion

This case highlights the potential risk of acute pancreatitis associated with the use of the GLP-1 RA, semaglutide. As aforementioned, GLP-1 RAs have gained popularity in the management of T2DM and obesity due to their efficacy in blood glucose regulation and weight management [12]. However, like any medication, GLP-1 RAs have side effects, predominantly GI in nature [8,13]. Liu et al. conducted a disproportionality study using the FDA Adverse Event Reporting System database (FAERS), analyzing 21,281 reports of GI toxicity [14]. They found that semaglutide had the greatest risk of GI side effects among GLP-1 RAs, with reported odds ratios (ROR) of 7.41 for nausea, 3.55 for diarrhea, 6.67 for vomiting, and 6.17 for constipation [14]. More concerning, however, is the potential risk of pancreatitis, which has been reported in several studies. Liu et al. demonstrated that semaglutide was associated with a significant risk of pancreatitis (ROR: 19.1) [14]. Additionally, Lando et al. noted increased serum amylase and lipase in 36% of diabetic patients started on GLP-1 RAs or dipeptidyl peptidase 4 (DDP4) inhibitors, compared to 18% in those not taking these medications [15]. These results demonstrate the presence of pancreatic inflammation in a significant proportion of patients started on incretin-based therapy [15]. 

It is important to note that the risk of pancreatitis associated with GLP-1 RAs is not limited to diabetic patients. Sodhi et al. reported a nine-fold increased risk of pancreatitis in non-diabetic obese subjects using GLP-1 agonists compared to those taking bupropion-naltrexone for weight loss [16]. This is particularly notable given the case of our young patient with acute pancreatitis, whereby the sole indication for semaglutide use was weight loss, as she did not have T2DM. 

The mechanism by which GLP-1 RAs may induce pancreatitis is not fully understood. However, Butler et al. observed in post-mortem examinations that patients treated with incretin therapy had an approximately 40% increase in pancreatic mass, with increased exocrine cell proliferation and dysplasia [17]. Endocrine cell changes, including α-cell hyperplasia, glucagon-expressing microadenomas, and a sixfold increase in β-cell mass, were also noted [17]. The intra- and periductal cell proliferation observed could potentially lead to obstruction of pancreatic enzyme outflow, thereby inducing pancreatitis [17].

While concerns have been raised about potential links between GLP-1 RAs and various forms of cancer, including pancreatic and thyroid cancer, recent evidence suggests these concerns may be unfounded. Nagendra et al. conducted a systematic review and meta-analysis involving 46,719 patients and found no association between semaglutide use and an increased risk of pancreatic, thyroid, or other cancers [18].

The case of our young patient, who developed acute pancreatitis within five weeks of exposure to semaglutide therapy, aligns with the findings of Singh et al. [19]. Their population-based case-control study involving 1,269 hospitalized cases of acute pancreatitis demonstrated that patients who were exposed to GLP-1 RAs within the last 30 days were over twice as likely to develop pancreatitis (adjusted OR: 2.24), while exposed within the past one to 24 months had a twofold increased risk (adjusted OR: 2.01) compared to non-users. Our patient had started semaglutide five weeks prior to presentation and was having weekly subcutaneous injections, thus the timing of her symptoms falls within this high-risk period indicated bySingh et al.

Acute pancreatitis is most commonly caused by gallstones or alcohol misuse, accounting for approximately 75% of cases. Other less common etiologies include hypertriglyceridemia, hypercalcemia, trauma, viral infections, autoimmune conditions, and medication-induced causes [20]. In our patient, these common causes were systematically excluded: she had undergone cholecystectomy 12 years ago, had no history of alcohol misuse, and reported no recent abdominal trauma, viral infections, or autoimmune disease symptoms. Laboratory workup was negative for leukocytosis, hypertriglyceridemia, hypercalcemia, and autoimmune antibodies, and imaging studies revealed no biliary pathology.

The recent initiation of weekly semaglutide five weeks before symptom onset, coupled with the absence of classic risk factors for acute pancreatitis, significant elevation in lipase levels, and negative imaging results, strongly suggests that subcutaneous semaglutide is the likely etiology of acute pancreatitis in this patient. Furthermore, upon discontinuation of semaglutide, the patient's lipase levels decreased from 1367 U/L to 99 U/L over a period of four days, indicating the resolution of pancreatitis. This rapid improvement following cessation of the medication further supports our diagnosis of acute pancreatitis likely due to semaglutide.

## Conclusions

This case highlights the need to raise awareness among patients taking semaglutide both prescribed and unprescribed, especially given its potential, though rare, association with acute pancreatitis. While GLP-1 receptor agonists like semaglutide are effective for managing T2DM and obesity, their role in triggering pancreatic inflammation remains incompletely understood. Immediate recognition of symptoms and cessation of the drug are crucial to prevent severe outcomes. This case underscores the importance of assessing the risk-benefit profile of semaglutide, particularly in patients with pancreatitis risk factors, and suggests the need for further research including further case reports and studies, to clarify the underlying mechanisms and guide safer clinical practices. In this scenario, the patient was unable to clarify the dose of semaglutide that she was taking as it was not prescribed; as a result, further research into whether the development of pancreatitis following semaglutide use is dose-dependent is also needed. Moreover, patients procuring semaglutide from non-medical professionals should be cautious about taking this medication without seeking medical advice.
